# Effect of Multispectral Pulsed Light-Emitting Diodes on the Growth, Photosynthetic and Antioxidant Response of Baby Leaf Lettuce (*Lactuca sativa* L.)

**DOI:** 10.3390/plants10040762

**Published:** 2021-04-13

**Authors:** Jurga Miliauskienė, Robert F. Karlicek, Elsebeth Kolmos

**Affiliations:** 1 Lithuanian Research Centre for Agriculture and Forestry, Institute of Horticulture, Kaunas str. 30, LT-54333 Babtai, Lithuania; 2 Rensselaer Polytechnic Institute, 110 8th Street, Troy, NY 12180, USA; karlir@rpi.edu (R.F.K.J.); kolmoe@rpi.edu (E.K.)

**Keywords:** LEDs, pulsed light, chlorophyll, phenols, antiradical activity

## Abstract

The effect of multicolor pulsed light-emitting diode (LED) irradiation on lettuce “Defender” growth, photosynthetic performance and antioxidant properties was studied. The experiments were designed to compare the continuous and pulsed lighting (0.5, 1 kHz; 50% duty ratio) effects of B450, G520, R660 and FR735 lighting components, maintaining total diurnal integral light quantity (DLI 14.4 mol m^−2^ day^−1^) constant during the 16-h photoperiod. The results showed that lettuce grown under pulsed irradiation displayed superior growth performance, including a significant enhancement of fresh (~32%) and dry biomass (~36%) and leaf area (~48%). Lettuce cultivated in both pulsed light treatments was characterized by the higher photosynthetic rate, chlorophyll (a,b) and carotenoid concentration. However, the total phenol and antioxidant properties in lettuce were more dependent on the specific pulsed light frequency. Only treatment with 1 kHz frequency was effective for higher phenol content, 2,20-azino-bis (3-ethylbenzothiazoline-6-sulphonic acid) (ABTS) free radical scavenging activity and Fe^2+^ reducing antioxidant power (FRAP). Thus, our results propose the role of pulsed LED light in improving the photosynthetic efficiency and antioxidative properties of lettuce plants cultivated indoors. In the future, pulsed lighting techniques should be included in the development of artificial lighting systems in controlled environment agriculture (CEA) to produce high-quality crops with the possibility to save electricity.

## 1. Introduction

Light is a crucial factor in the life cycle of plants and its quality and quantity, as well as the photoperiod, have significant effects on plant growth, development and metabolism. Manipulation of light for plant cultivation is an important issue that is studied in controlled environment agriculture (CEA), where several different artificial lighting conditions are commonly used in plant factories, growth chambers and greenhouses. Light-emitting diode (LED)-based horticultural lighting systems have increasingly been used in CEA to research the physiological responses of plants to light as well as for commercial production of horticultural crops [1]. LED lighting is more energy efficient compared to the traditional high-pressure sodium or fluorescent lighting used in horticulture and allows the light spectrum, intensity and duration to be tailored to enhance specific physiological responses in plants and to optimize the light use efficiency when cultivating high-value crops [2,3,4]. Numerous studies have investigated and reviewed the effects of LED lighting quality on plants [5,6]. Notably, blue and red LED light combinations are most commonly used, as these wavelengths are predominantly absorbed by photosynthetic pigments and are sufficiently effective for plant growth and photosynthesis of various crops [6,7,8]. Other LED wavelengths, like green, far-red and UV-A, have remarkably lower photon efficacies, but in recent years, the remarkable effect of supplemental light colors has been explored [6]. Therefore, in closed CEA systems, where artificial lighting is the only source of light, the lighting strategies have changed from simple red and blue combinations to a multicomponent lighting spectrum, mimicking the wide spectrum of the sun.

In the plant production process, the economic aspects of lighting are of major importance. It is reported that the electricity cost in plant production accounts for about 25% of the total production costs [9,10]. Thus, measures to improve the efficiency of electricity and light use, both by improving the performance of LED light source and by harmonizing plant physiological needs for the quality and the economic aspects of lighting would be desirable. During recent years, research on LEDs has yielded technologies that make LED modules more energetically efficient and versatile as plant lighting. LEDs can be used for the application of pulsed irradiation to plants. The development of LED modules and controllers has made it possible to explore a vast parameter space including frequency, duty factor and wavelength of light. The main objective of pulsed LED lighting is to lower the electric energy cost without compromising yield and crop quality. The pulse irradiation is generated using a pulse width modulation technique consisting of light and dark periods operating at various frequencies. Because plants can grow well with seconds-long pulses rather than continuous exposure, it becomes possible to explore energy savings by extending the dark period between pulses (duty factor) [11]. The hypothesis is that a defined frequency of pulses may lead to production of identical plant products with fewer photons invested. Only a few reports on the effects of pulsing LED light on plant growth, photochemistry and nutritional quality have been published. The results [1] demonstrated that pulsed LED treatment with a 75% duty factor has no inhibition on the growth of lettuce as compared to continuous light, and a pulsed light treatment at 1 kHz was the most effective in terms of growth as well as energy use efficiency. Furthermore, pulsed light at high frequencies (2–20 kHz, 50% duty factor) positively affects the growth of lettuce under controlled environmental conditions [12]. The pulsing ability of flexible LED lighting systems to produce sufficient photon fluxes of specific wavelengths can be linked with characteristics of photosynthesis and satisfy the photosynthesis requirements [13]. Several studies were reported on pulsed LED lighting effects on photochemical efficiency of photosynthesis; a better quantum efficiency of photosystem II (PSII) under pulsed LEDs (50% ratio with 0.1, 1, 100 or 1000 Hz) was found for tomato plants [14]; and significantly higher quantum efficiency of PSII and photosynthetic electron transport was found for wheat [15]. However, when comparing intermittent vs. continuous light, no significant effects on quantum efficiency of photosystem II in lettuce plants was found in the results by Son et al. [1]. In the literature, the effects of pulsed lighting on the secondary metabolism of plants were rarely demonstrated. Pulsed LEDs were tested on *Brassica* microgreens in order to achieve a higher nutritional level [13]. The pulsed lighting resulted in induced secondary metabolic reactions and initiated phytochemical changes in the microgreens, where the effect depended upon the pulse frequencies of applied LED light wavelengths and varied among microgreen species [13]. Dong et al. [15] showed a comparable result of intermittent light vs. continuous lighting when the antioxidant capacity of wheat plants was analyzed.

In recent decades, pulsed irradiation to improve the efficiency of crop incident light flux was discussed, but there is still no general consensus on the benefits of pulsing light for plant growth. An array of plant species, spectral combinations and intensities, photoperiods and different frequencies and duty ratios have been used in the literature. However, little data available on the effect of pulsed LEDs make it difficult to draw a general conclusion on, e.g., the growth and secondary metabolism of lettuce, which is a popular crop in CEA. Therefore, the objective of this study was to determine the growth, leaf photosynthetic and antioxidative responses of the popular CEA crop lettuce following growth under pulsed LED light in comparison to continuous light with the same daily light integral (DLI).

## 2. Results

**Figure 1:**
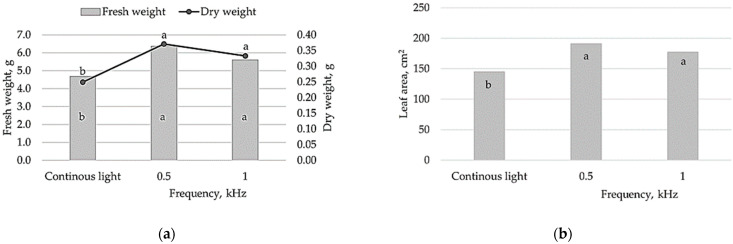
Effect of continuous lighting and different pulsed lighting (0.5, 1 kHz) on lettuce growth characteristics: (**a**) fresh and dry weight; (**b**) leaf area. Different letters represent significant differences. The data were processed using analysis of variance (ANOVA), the Tukey (HSD) multiple range test at the confidence level *p* = 0.05.

The research results confirmed that pulsed vs. continuous lighting had pronounced effects on lettuce growth, photosynthesis and antioxidative properties. In lettuce, pulsed lighting at different frequencies significantly increased fresh and dry weight as well as leaf area compared to continuous lighting (Figure 1). No significant differences in the measured growth parameters were observed for plants treated with 0.5 or 1 kHz pulsed light flux; however, the lower light pulse fluxes (0.5 kHz) tended to increase lettuce biomass and leaf area.

**Figure 2:**
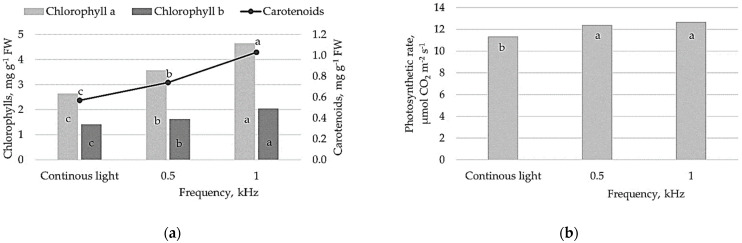
Effect of continuous lighting and different pulsed lighting (0.5, 1 kHz) on chlorophyll a, b and carotenoids content (**a**) and net photosynthetic rate (**b**) of lettuce. Different letters represent significant differences. The data were processed using analysis of variance (ANOVA), the Tukey (HSD) multiple range test at the confidence level *p* = 0.05.

The significantly higher content of photosynthetic pigments, chlorophyll a and b (Chla, Chlb) and carotenoids were determined in lettuce illuminated by pulsed light flux compared to plants grown under continuous light (Figure 2a). Furthermore, the content of Chla, Chlb and carotenoids differed between pulsed lighting treatment significantly; 1 kHz lighting resulted in 1.3- and 1.4-fold higher content of chlorophylls and carotenoids in lettuce leaves, respectively.

Pulsed light treatment resulted in a significant increase of the photosynthetic rate (Pn, µmol CO_2_ m^−2^ s^−1^) in lettuce plants. The measured values show that Pn was 10% and 12% higher under 0.5 and 1 kHz pulsed light treatments, respectively (Figure 2b).

The content of total phenolic compounds differed between constant and pulsed lighting treatment significantly (Table 1).

The highest content of total phenolics was determined in lettuce subjected to 1 kHz pulsed light, while the lowest one was for plants grown under 0.5 kHz pulsed flux or continuous lighting. The antioxidant properties of lettuce measured by several methods showed different responses of antioxidative activity to continuous and pulsed light treatments. No significant differences in 2-diphenyl-1-picrylhydrazyl (DPPH) free radical scavenging activity in lettuce were observed (Table 1). The strongest 2,20-azino-bis (3-ethylbenzothiazoline-6-sulphonic acid) (ABTS) free radical scavenging activity was determined in plants subjected to 1 kHz pulsed light flux. Fe^2+^ reducing antioxidant power (FRAP) was determined to be significantly different between continuous and pulsed light treatments (Table 1). The 1 kHz pulsed light flux resulted in a significantly higher FRAP value compared to other treatments. The lowest FRAP value was determined for plants subjected to 0.5 kHz pulsed light flux.

**Figure 3:**
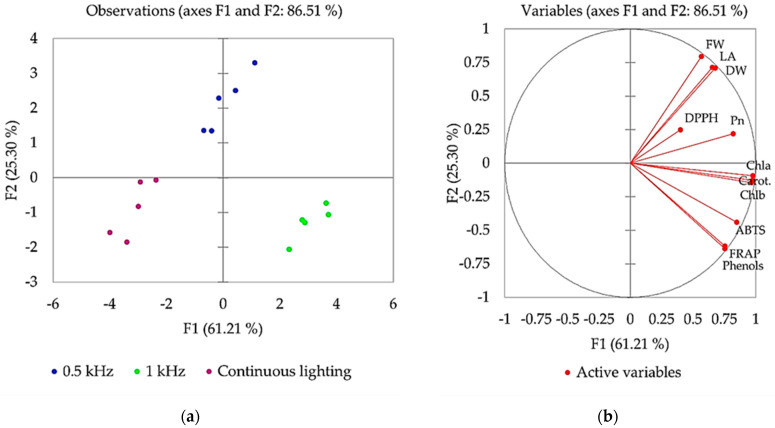
Principal component analysis (PCA) (**a**) and correlation circle (**b**) demonstrating the growth and physiological responses of lettuce subjected to continuous and different pulsed (0.5, 1 kHz) lighting. FW, fresh weight; DW, dry weight; LA, leaf area; Pn, net photosynthetic rate; Chl a, chlorophyll a; Chl b, chlorophyll b; Carot, carotenoids; Phenols, total phenolic compounds; DPPH, 2-diphenyl-1-picrylhydrazyl radical scavenging activity; ABTS, (2,20-azino-bis (3-ethylbenzothiazoline-6-sulphonic acid)) diammonium salt radical scavenging activity; FRAP, Fe^2+^ reducing antioxidant power assay.

The principal component analysis (PCA) was performed to obtain relationships between the growth and physiological parameters in the response to different light treatments. The first two factors (F1 vs. F2) of the PCA, as shown in the scatterplot (Figure 3a) and correlation circle (Figure 3b), explained 86.51% of the total variance of the data set.

The loading data reveal a clear separation between pulsed and continuous light-treated plants, indicating a distinct pattern of lettuce growth in response to the different source of light. The F1 components, which comprised 61.21% of the total variance of the data set, clearly separated 1 kHz light treatment from the continuous light treatment. This variation was mainly attributable to the positive loadings of parameters such as chlorophylls a and b, carotenoids, photosynthetic rate, total phenolic compounds and antioxidant properties (ABTS, FRAP). Along the F2 component, which explained about 25.30% of the total variance of the data set, a distinct separation was observed in the plants grown under 0.5 kHz light. This separation was mainly driven by the positive loadings of parameters such as leaf area, fresh and dry weight. Figure 3b demonstrates the correlation between different variables measured in lettuce, where two vectors with an angle less than 90° are positively correlated and two vectors with an angle higher than 90° are negatively correlated. A strong positive correlation between leaf area and fresh and dry weight and a moderate correlation between photosynthetic pigments, photosynthetic rate and leaf area and biomass accumulation was found (Figure 3b). There was a very strong or strong positive correlation between Chla, Chlb, carotenoids and phenolic compounds (Figure 3b). In terms of antioxidative properties, a strong positive correlation was determined between ABTS and FRAP, and total phenolic compounds were highly related to these properties (Figure 3b).

## 3. Discussion

Leafy green vegetables, such as lettuce and baby leaf lettuce, are dominant crops in CEA. The growth and quality of leafy greens can be purposefully tailored by adjusting environmental parameters, including artificial lighting [9,16]. Regarding pulsed illumination, various studies in the literature confirm different plant growth responses to pulsed lighting and this usually depends on pulsed light parameters such as frequencies, duty ratios and photosynthetic photon flux density (PPFD). The results described above, as well as the PCA analysis, confirm that lettuce differentially responded to continuous and pulsed illumination with the same DLI. In general, we observed that lettuce growing under pulsed light conditions displayed superior growth performance, increased photosynthetic pigment synthesis and improved photosynthesis. However, the total phenol content and antioxidant properties in lettuce were dependent on the applied pulsed light frequency.

Thus, in this study, both pulsed light treatments tested (0.5 and 1 kHz) had distinct impact on lettuce biomass accumulation compared to continuous lighting. The growth parameters of lettuce, in terms of assimilating leaf area, fresh and dry biomass showed up to 32%, 36% and 48% higher values in the pulsed light treatments, respectively (Figure 1). A strong positive correlation suggests that pulsed light-mediated changes in lettuce biomass accumulation could be related to increased leaf area and therefore more efficient light interception (Figure 3b, Table S1). In agreement with our results, several reports have shown a positive effect of pulsed light on lettuce fresh weight and photosynthetic activity [17], including the morphological and physiological response of carnation [18]. In contrast, other studies revealed that lettuce growth did not differ significantly when grown under pulsed LEDs with a range of frequencies (0.3, 1, 3, 10 and 30 kHz) and 75% duty ratio; however, this was explained by the difference in PPFD [1]. Pulsed light with lower PPFD has a lower light intensity and thus a lower amount of light energy that can negatively affect plant growth and development. The data suggest [1] that pulsed LED irradiation must have sufficient PPFD for effective photosynthesis performance in plants, and duty ratios and frequencies are the key factors of light intensity in pulsed treatments. Our results suggest that pulsed irradiation at 0.5 and 1 kHz frequencies at 50% duty ratio (PPFD 250 μmol m^−2^s^−1^) could be more effective for lettuce biomass production compared to continuous light.

Due to the photochemical nature of photosynthesis, the photosynthetic rate, which represents the amount of O_2_ production or the amount of CO_2_ fixation per unit time, correlates well with the number of photons reaching the surface per unit area per second. An efficient light interception under pulsed light promoted photochemical reactions in the lettuce and resulted in efficient photosynthesis compared to continuous lighting (Figure 2b). Light-harvesting complexes with pigments are crucial for photosynthetic efficiency, and photosynthetic pigment content is an important factor for plant growth and development, which is controlled by different light qualities [19]. Both red/far-red light photoreceptors (phytochromes) and blue light photoreceptors (cryptochromes) mediate the light signaling, which modulates the expression of genes involved in chlorophyll metabolism. In the present study, we found that pulsed lighting significantly affected the synthesis and accumulation of Chla, Chlb and carotenoids (Figure 2a), suggesting that the chlorophyll biosynthesis pathway and cycle are induced by pulsed lighting. Here, we can presume that pulsed red/far-red and blue LED irradiation has led to enhanced 5-aminolevulinic acid (5-ALA) synthesis activity and the upregulation of gene expression encoding enzymes (Mg-chelatase, Fe-chelatase, GluTR, etc.), which are involved in chlorophyll biosynthesis pathway [19,20]. In the lettuce plants, the higher content of photosynthetic pigments led to an increase in light absorption and reactive oxygen species removal from plants in the presence of pulsed light, resulting in more efficient photosynthesis and ultimately contributing to remarkable biomass growth. The positive correlation between photosynthetic performance and plant biomass (Figure 3b, Table S1) suggests that the higher photosynthetic rate in lettuce illuminated by pulsed light had a remarkable effect on biomass accumulation, indicating that pulsed light acts as an equitable signal for photochemical reactions in lettuce plants. Previous studies estimated that the photosynthetic rate for various crops responded differently to pulsed lighting and highly depended on the frequency and duty ratio of pulsed light [21]. For instance, Jishi et al. [22] explored the effects of pulsed LEDs at different frequencies (0.1, 0.2, 0.4, 0.8, 1.6, 3.2, 6.4, 12.8 kHz; 75% duty ratio) on romaine lettuce and did not find a significant effect on net photosynthesis compared to continuous light. Kanechi [12] found that photosynthetic rate of lettuce under pulsed light (0.5–500 Hz, 1–20 kHz, 50% duty ratio) was slightly higher than that under continuous light, and this is consistent with our results. The observed effects of pulsed-light parameters on net photosynthesis could be explained by the pooling of photosynthetic intermediates [23]. Chloroplasts are expected to pool photosynthetic intermediates during the light period using the reducing power derived from the oxidation of water and subsequently consume the photosynthetic intermediates to fix CO_2_ during the dark period. In this study, relative to continuous light, the pulse frequencies at 0.5 and 1 kHz and 50% duty ratio had to be optimal for lettuce growth, inferring that pulsed illumination with such a condition is more advantageous than continuous light for efficient photosynthesis leading to improved biomass production. This phenomenon can be explained by optimized light capture and light energy conversion in the light reactions, and carbon capture and conversion in the dark reaction, which together are important for whole photosynthetic efficiency and biomass production.

Any change in light quality affects plant physiological and biochemical properties [19]. Therefore, understanding the plant response to pulsed light is of practical importance not only for plant growth but also for the nutritional quality. Moreover, certain lighting parameters are important in regulating plant metabolism and phytochemical accumulation. In the present study, pulsed LED light evoked the response of secondary metabolism and initiated the phytochemical changes in lettuce tissue. The effect of pulsed light depended on the light frequency and was more pronounced in lettuce subjected to 1 kHz rather than 0.5 kHz (Table 1). We found that lettuce exposed to 1 kHz accumulated significantly higher content of total phenolic compounds (Table 1). Due to the ability to scavenge free radicals, phenolic compounds are important contributing factors to antioxidant activity. This result was supported by significant correlations between total phenolic compounds and ABTS radical scavenging activity and FRAP antioxidant power (Figure 3b), clearly indicating that pulsed light (1 kHz) stimulates the phenylpropanoid pathway for the synthesis of phenolic compounds in lettuce. Our results are consistent with previous results reported that pulsed light positively affects the total phenolic compounds (2, 256 and 1024 Hz) and antiradical activity (32, 256 and 1024 Hz) in mustard [13]. However, there are not many data available on the effects of artificial pulsed LED light on polyphenols and the antioxidant capacity of plants, and the mechanisms by which these antioxidative properties are enhanced by pulsed LED light remain unclear. Based on available data regarding polyphenols [24,25,26], the increase in total phenolic compounds in pulsed light exposed lettuce could be due to the stimulating effect of pulsed red and blue light on the level of gene expression and activity rates of the key enzymes involved in the biosynthesis pathway of polyphenols. Key enzymes related to the phenylpropanoid pathway, phenylalanine ammonia-lyase (pal) and chalcone synthase (chs) are associated with polyphenol biosynthesis in plants [25]. These enzymes play a major role in plant development and defense mechanisms [26]. Though pal is the first enzyme in the phenylpropanoid pathway inducing the biosynthesis of polyphenols, in the present study chs could have a favorable role on phenolic compound biosynthesis in lettuce illuminated by pulsed light (1 kHz frequency). Previous studies have shown that *chs* gene can be induced by abiotic stress such as light/UV light or by circadian rhythms and is closely associated with the dark/light cycle [25]. In addition, chlorophylls and carotenoids may play a significant role in increased antioxidant activity in lettuce, as positive correlation was found between chlorophylls and carotenoid content and antioxidant activity (Figure 3b, Table S1). Chloroplasts contain many different antioxidants, including chlorophylls and carotenoids that often have overlapping or interacting functions. The function of chloroplast antioxidants is to achieve an appropriate balance between reduction–oxidation reactions, compatible with the photosynthetic operation, and allowing these redox signals to be efficiently transmitted to the nucleus [27].

## 4. Materials and Methods

### 4.1. Plant Material and Growth Conditions

Experiments were performed in controlled environment plant growth chambers CONVIRON Adaptis (model A1000, Conviron, Winnipeg, Manitoba, Canada) at Rensselaer Polytechnic Institute (RPI), Lighting Enabled System and Application Center (LESA), USA. Seeds of green romaine lettuce “Defender” (*Lactuca sativa* L.; Johnny’s Selected Seeds, Winslow, ME, USA) were sown in rockwool cubes (2.5 cm × 2.5 cm× 3.0 cm) presoaked in deionized water and placed in plastic trays (30 cm × 50 cm). Seedlings were germinated in growth chambers and were subjected to continuous LED lighting (Tunable Irradiant Growth Efficacy Research (TIGER) light, RPI, USA), which included a combination of blue (B, λ = 450 nm, 15%), green (G, 520 nm, 10%), red (R, λ = 660 nm, 75%) and additionally far-red (FR, λ = 735 nm, 10%) LED; the PPFD was approximately 250 μmol m^−2^s^−1^ in total. The environmental conditions were: 21 ± 2/18 ± 2 °C day/night temperatures, 50–60% relative humidity and a 16 h photoperiod. After 5 days, a customized Hoagland’s solution [28] was supplied in each culture tray with the following average concentration of nutrients (mg L^−1^): N, 190; P, 50; K, 200; Ca, 45; Mg, 80; S, 106; Fe, 2; Mn, 0.2; Cu, 0.1; B, 0.4; Zn, 0.2; Na, 1; Mo, 0.08. The pH and the electrical conductivity (EC) of the nutrient solution were measured daily using a portable meter (Bluelab commercial Truncheon^®^ meter, Bluelab Corporation Limited, New Zealand). The nutrient solution was renewed every week and adjusted to pH 6 and an EC of 1.4 mS cm^−1^. For each treatment, 78 seedlings were placed in a tray and were treated with irradiation from pulsed LEDs for 3 weeks under the same growth conditions as described above.

### 4.2. Pulsed Lighting Treatments

The TIGER LED lighting system was designed and built by LESA at RPI, USA. This system is based on a custom 6-in-1 LED package (Prolight Optoelectronics, Taiwan) where six separate LED chips are tightly arranged in a single package with a single lens for excellent color mixing. In this package, the six separate wavelengths were: violet (V, peak wavelengths 400 nm), indigo (I, peak wavelengths 420 nm), blue (B, peak wavelengths 450 nm), green (G, peak wavelengths 520 nm), red (R, peak wavelengths 660 nm), far-red (FR, peak wavelengths 735 nm) (Table 2).

The TIGER light is able to emit a pulsed light for each wavelength in any pattern–synchronous, phase shifted or different frequencies for each wavelength. The TIGER light system utilized a simple high-speed LED driver system for both continuous wave and pulsed operation. Pulsing sequences were selected from a user lighting recipe file and downloaded to a Raspberry PI controller that executed required lighting protocol, including intensity per color, pulse duration (and duty factor) per color and desired lighting interval over the course of 24 h. The peak fluence per color channel is shown in Table 2, measured at a distance of 44 cm from the TIGER lighting fixture. Intensity uniformity over the area of the growth area of the Conviron cabinet was measured to be +/−5%.

The initial experiments were designed to compare the continuous and pulsed lighting effects of B450, G520, R660 and FR735 lighting components, maintaining total diurnal integral light quantity (daily light integral, DLI) constant during the 16-h photoperiod (Table 3). Three lighting treatments were designed: constant flux of B450, G520, R660 and FR735 (continuous); synchronous pulsed flux of B450, G520, R660 and FR735 at different frequencies (0.5; 1 kHz) with 50% duty ratio. At the end of the growing period, biometric, photosynthetic and biochemical analyses were performed. All biochemical analysis was performed in at least five biological replications.

### 4.3. Growth Characteristics

After 3 weeks of treatment, the shoot weight (fresh and dry), and leaf area were measured for 10 plants per treatment (*n* = 10). The fresh biomass was measured with an electronic scale (Mettler Toledo, ML104T/00; Mettler-Toledo, Columbus, OH, USA) and subsequently the shoots were dried at 70 °C in an oven for 2 days before measuring dry weight. The leaf area was measured using a leaf area meter (CI-202 Laser Area Meter; CID BioScience, Camas, WA, USA).

### 4.4. Photosynthesis

The amounts of chlorophyll and carotenoid in green leaves were determined by spectrophotometry in absolute acetone extract [29]. Leaf samples were ground in liquid nitrogen and homogenized in pure iced acetone, then incubated for 24 h at 4 °C. Next, 400 µL of supernatant was diluted up to 1000 µL using pure acetone. The absorbance of each sample was read with a spectrophotometer (Jasco V-570; JASCO Corporation, Tokyo, Japan) at 470, 663.2 and 646.8 nm against a pure acetone blank. The concentration of chlorophyll a, chlorophyll b and carotenoids was calculated using the following equations [30]:

Chlorophyll a = 12.25A_663.2_ − 2.79A_646.8_ (1)

Chlorophyll b = 21.50A_646.8_ − 5.10A_663.2_ (2)

Carotenoids = (1000A_470_ − 1.82Chla − 85.02Chlb)/198 (3)

Net photosynthetic rate (Pn, µmol CO_2_ m^−2^ s^−1^) was measured using a portable photosynthesis system Ciras-3 (PP Systems, Amesbury, MA, USA) on randomly selected, youngest, fully-expanded leaves between 09:00 and 12:00. The CO_2_ concentration within the leaf cuvette was maintained at 390 ± 10 mol mol^−1^, temperature was set at 21 °C. Air flow rate through the assimilation chamber was set at 300 µmol s^−1^; photosynthetically active radiation at the leaf surface was set depending on the individual treatment. After being embedded in the assimilation chamber, the leaves were allowed to acclimate until the gas exchange was in a steady state. The Pn measurements were performed on at least five plants per treatment.

### 4.5. Antioxidant Properties and Total Phenolic Compounds

Antioxidant properties of lettuce leaves were evaluated as the 2-diphenyl-1-picrylhydrazyl DPPH, (2,20-azino-bis (3-ethylbenzothiazoline-6-sulphonic acid)) diammonium salt radical scavenging activities (ABTS) and Fe^2+^ reducing antioxidant power assay (FRAP); also, total contents of phenolic compounds were determined [31]. Extracts were prepared by grinding plant material with liquid nitrogen and diluting with 80% methanol 1:10 (*w*:*v*). After 24 h, extracts were filtered through cellulose filters. The DPPH free radical scavenging activity was determined by mixing the diluted extract with 0.06 M methanolic DPPH solution, and radical quenching, monitored every minute for 16 min, measuring absorbance at 515 nm (Jasco V-570; JASCO Corporation, Tokyo, Japan). The ability of plant extract to scavenge DPPH free radicals was calculated using the DPPH solution as a blank. The results are presented as DPPH free radical scavenging activity, µmol g^−1^ of fresh plant weight.

The ABTS radical solution was prepared by mixing 50 mL of 2 mM ABTS with 200 µL 70 mM K_2_S_2_O_8_, allowing the mixture to stand in the dark at room temperature for 16 h before use. The working solution was diluted to obtain initial absorbance of AU 0.700 at 734 nm (Jasco V-570; JASCO Corporation, Tokyo, Japan). Then, 100 µL of the sample was mixed with 2 mL ABTS solution, and absorbance was monitored for 11 min. The ability of plant extract to scavenge ABTS free radicals was calculated using the ABTS solution as a blank. The results are presented as ABTS free radical scavenging activity, µmol g^−1^ of fresh plant weight. FRAP method was based on reducing ferric ion (Fe^3+^) to ferrous ion (Fe^2+^). FRAP working reagent was prepared by mixing acetate buffer (300 mM, pH 3.6), a solution of 10 mM 2,4,6-tripyridyl-s-triazine (TPTZ) in 40 mM HCl, and 20 mM FeCl_3_ × 6H_2_O at 10:1:1 (*v*/*v*/*v*). Next, 20 µL of the sample was mixed with 3 mL of working solution and incubated in the dark for 30 min. Then, absorbance at 593 was read. The antioxidant power calculation was performed using Trolox calibration curve and was expressed as Trolox equivalent antioxidant capacity (TEAC, µmol Trolox per g^−1^ of fresh plant weight).

The total content of phenolic compounds of lettuce leaves was determined using Folin–Ciocalteu reagent [32]. First, 250 µL aliquot of the sample extract was mixed with 250 µL of 10% (*w*/*v*) Folin–Ciocalteu reagent, 500 µL of 1 M Na_2_CO_3_ solution and 2 mL of distilled water. After incubation for 20 min in the dark, the absorbance at 765 nm was read in a spectrophotometer (Jasco V-570; JASCO Corporation, Tokyo, Japan). The results were expressed in mg of gallic acid equivalents per g of fresh weight of plant tissues.

### 4.6. Statistical Analysis

The data were processed using XLStat software (Addinsoft, USA, 2019). Means were statistically tested using analysis of variance (ANOVA) along with the post-hoc Tukey’s HSD test. Differences were considered to be significant at *p* = 0.05. Multivariate principal component analysis (PCA) was performed using XLStat. The results are presented in a PCA scatter plot showing the distinct differences in the growth and physiological response of lettuce under different lighting characteristics, and a correlation circle (based on Pearson’s correlation matrix) summarizes the relationships between the investigated parameters.

## 5. Conclusions

In this research, lettuce plants responded differently to multicolor LED lighting when it was continuous or pulsed light at 0.5 and 1 kHz frequency. Generally, our experiments and research results show that pulsed LED lighting significantly promotes plant growth in terms of leaf area and fresh and dry weight. The pulsed light conditions influenced the biosynthesis of photosynthetic pigments and the activity of photosynthetic rate. Furthermore, we determined that exposure to the specific frequency of LED light pulses can enhance the accumulation of secondary metabolites and the antioxidant properties of lettuce plants. Undoubtedly, further research is needed to elucidate the physiological responses of plants exposed to pulsed LED light; however, our results propose the role of pulsed LED light in improving photosynthetic efficiency and antioxidative properties of lettuce plants. Pulsed lighting techniques are quite an innovative technology; therefore, it is important to determine the optimal frequency and duty ratio for plants to attain the most efficient use of harvested light. High-speed pulsing capabilities should be included in the development of artificial horticulture lighting systems as an important consideration for future CEA lighting systems, in order to be able to produce high-quality crops.

## Figures and Tables

**Table 1:** Effect of continuous lighting and different pulsed lighting (0.5, 1 kHz) on total phenolic content and antioxidant properties of lettuce. Different letters represent significant differences. The data were processed using analysis of variance (ANOVA), the Tukey (HSD) multiple range test at the confidence level *p* = 0.05.

Frequency, kHz	Total Phenols, mg g^−1^ FW	DPPH, μmol g^−1^ FW	ABTS, μmol g^−1^ FW	FRAP, μmol Trolox g^−1^ FW
Continuous lighting	0.33 b	4.23 a	25.97 b	168.92 b
0.5	0.29 b	4.46 a	27.56 b	152.87 c
1	0.56 a	4.41 a	39.13 a	251.32 a

**Table 2:** Wavelengths and fluence for each of the TIGER channels.

Color.	λ (nm)	μmol m^−2^s^−1^
violet	400	375
indigo	420	458
blue	450	634
green	520	279
red	660	594
far-red	735	378

**Table 3:** Experimental design.

Lighting Treatment	% of Total PPFD, μmol m^−2^s^−1^	Total DLI, mol m^−2^ day^−1^	Duty Ratio, %
Continuous	R 75%, B 15%, G 10%, FR 10%	14.4	100
0.5 kHz			50
1 kHz			

## Data Availability

Not applicable.

## Supplementary Materials

The following are available online at https://www.mdpi.com/article/10.3390/plants10040762/s1, Table S1: The correlation matrix (Pearson (n)) between growth indices, photosynthetic and antioxidant properties in lettuce.
