# Supplementary material for: Effect of Multispectral Pulsed Light-Emitting Diodes on the Growth, Photosynthetic and Antioxidant Response of Baby Leaf Lettuce (Lactuca sativa L.)
Source: Plants (Basel). 2021 Apr 13;10(4):762. doi: 10.3390/plants10040762 (PMC8069217; doi:10.3390/plants10040762)
Supplement: Supplementary file 1 [file plants-10-00762-s001.pdf]

plants

Supplementary Materials:

Table S1. The correlation matrix (Pearson (n)) between growth indices, photosynthetic and antioxidant properties in lettuce.

Variables	LA	FW	DW	Pn	Chl a	Chl b	Carot	Phenols	DPPH	ABTS	FRAP
LA	**1**	**0.976**	**0.959**	**0.656**	**0.599**	**0.557**	**0.576**	0.067	0.324	0.276	0.078
FW	**0.976**	**1**	**0.965**	**0.579**	0.488	0.443	0.460	-0.066	0.266	0.128	-0.048
DW	**0.959**	**0.965**	**1**	**0.632**	**0.576**	**0.529**	**0.553**	0.047	0.313	0.238	0.084
Pn	**0.656**	**0.579**	**0.632**	**1**	**0.709**	**0.684**	**0.704**	0.450	**0.572**	**0.624**	0.506
Chl a	**0.599**	0.488	**0.576**	**0.709**	**1**	**0.995**	**0.999**	**0.792**	0.315	**0.842**	**0.773**
Chl b	**0.557**	0.443	**0.529**	**0.684**	**0.995**	**1**	**0.994**	**0.819**	0.312	**0.855**	**0.796**
Carot	**0.576**	0.460	**0.553**	**0.704**	**0.999**	**0.994**	**1**	**0.812**	0.291	**0.857**	**0.798**
Phenols	0.067	-0.066	0.047	0.450	**0.792**	**0.819**	**0.812**	**1**	0.136	**0.908**	**0.974**
DPPH	0.324	0.266	0.313	**0.572**	0.315	0.312	0.291	0.136	**1**	0.225	0.081
ABTS	0.276	0.128	0.238	**0.624**	**0.842**	**0.855**	**0.857**	**0.908**	0.225	**1**	**0.899**
FRAP	0.078	-0.048	0.084	0.506	**0.773**	**0.796**	**0.798**	**0.974**	0.081	**0.899**	**1**

Values in bold are different from 0 with a significance level alpha=0.05. LA, leaf area; FW, fresh weight; DW, dry weight; Pn, net phtososynthetic rate; Chl a, chlorophyll a; Chl b, chlorophyll b; Carot, carotenoid; Phenols, total phenolic compounds; DPPH, 2-diphenyl-1-picrylhydrazyl radical scavenging activity; ABTS, (2,20 -azino-bis (3-ethylbenzothiazoline-6-sulphonic acid)) diammonium salt radical scavenging activity; FRAP, Fe^{2+} reducing antioxidant power assay.
